# Evaluation of a real-time method of simultaneous amplification and testing in diagnosis of *Mycoplasma pneumoniae* infection in children with pneumonia

**DOI:** 10.1371/journal.pone.0177842

**Published:** 2017-05-16

**Authors:** Wei Li, You-hong Fang, Hong-qiang Shen, De-hua Yang, Qiang Shu, Shi-qiang Shang

**Affiliations:** Department of Clinical Laboratory, Children’s Hospital of Zhejiang University School of Medicine, Hangzhou, PR China; Miami University, UNITED STATES

## Abstract

*Mycoplasma pneumoniae* (*M*. *pneumoniae*) infection can cause community acquired pneumonia in children. A real-time method of simultaneous amplification and testing of *M*. *pneumoniae* (SAT-MP) was developed to diagnose *M*. *pneumoniae* targeting a region of the ribosomal RNA. The SAT-MP assay can accurately identify *M*. *pneumoniae* with a detection range from 10^1^ to 10^7^ CFU/ml. In this study, the specimens from 315 children with pneumonia were collected and analyzed by SAT-MP in parallel with real-time PCR method and IgM ELISA assay. The positive rates of these specimens examined by SAT-MP assay, real-time PCR method and IgM ELISA assay were 16.51%, 15.56% and 12.70% respectively. While there was statistical significance (p = 0.04) between SAT-MP assay and IgM ELISA assay, no statistical significance (p = 0.25) was found between SAT-MP assay and real-time PCR method and these two methods had high consistency (kappa value = 0.97). These findings indicate that the newly developed SAT-MP assay is a rapid, sensitive and specific method for identifying *M*. *pneumoniae* with potential clinical application in the early diagnosis of *M*. *pneumoniae* infection.

## Introduction

*M*. *pneumoniae* is recognized as one of the most common pathogens causing community-acquired pneumonia (CAP) in certain populations, especially in children [1,2]. Moreover, in children, infection can lead to severe pneumonia requiring hospitalization [3]. Our previous studies showed that the main population infected with *M*. *pneumoniae*was older children [4, 5]. Therefore, early and rapid detection of *M*. *pneumoniae* is important in clinical treatment.

Currently, serological tests, real-time PCR and Loop-Mediated Isothermal Amplification (LAMP) are well-established and widespread diagnostic methods for rapid diagnosis of *M*. *pneumoniae* [6–11], replacing time-consuming and less sensitive culture methods in clinical practice [6,7]. However, antibodies to *M*. *pneumoniae* may not appear until 2 weeks following the onset of symptoms, restricting the utility of serological tests in early diagnosis [8]. And real-time PCR is incapable of distinguishing live bacteria from the dead ones. Given the drawbacks of current tests, novel rapid diagnostic techniques with high sensitivity and specificity are required for early identification of *M*. *pneumoniae* infections. Simultaneous amplification and testing(SAT) is a recently developed method based on isothermal amplification of RNA and real-time detection of fluorescence which was previously established to detect *Mycobacterium tuberculosis* and hepatitis C virus [12–14]. The aim of this study was to establish and evaluate potential utility of SAT for early detection of *M*. *pneumoniae* in clinical practice.

## Methods

### Patients

From January 2016 to April 2016, a total of 315 children were enrolled in this study, including 202 boys and 113 girls. The ages of the study participants ranged from 1 month to 10 years. All study participants had been primarily diagnosed with pneumonia [15] and had received no clinical treatment. During study period, two respiratory tract samples (throat swab or sputum) from every child were collected. One sample was mixed with 1 ml normal saline for real-time PCR and the other was mixed with 500μl RNA protective agent (RNase inhibitor and lysis reagent) for SAT-MP.

### Ethics statement

This study was approved by the ethics committee of the Children’s Hospital, Zhejiang University School of Medicine, and written consent was obtained from patients’ parents or legal guardians.

### Serological test for detection of IgM antibody of *M*. *pneumoniae*

Blood samples and respiratory tract samples were collected at the same time. The serum was separated and then stored at -70°C till the time of analysis. The *M*. *pneumoniae* IgM was determined via a specific Anti-*M*. *pneumoniae* ELISA assay (EUROIMMUN, Luebeck, Germany), the absorbance above 1.1 was determined positive. All performance was according to the manufacturer's instructions.

### Real-time PCR for detection of *M*. *pneumoniae*

The primers and probe were reported in previous study which target the p1 gene of *M*. *pneumoniae* [16]. 1 ml of the mixture containing sample and normal saline was moved into a 1.5-ml microcentrifuge tube aseptically and centrifuged for 5 min. The supernatant was decanted and the cell pellets were suspended in 50μl of lysis buffer (Da’an gene company, China). And the mixture was then boiled for 10 min. 4μl of supernatant was served as the template in real-time PCR system, containing 400 nM of each forward and reverse primer, 100 nM of fluorescently-labeled specific probe(5’HEX—3’BHQ1), and 25μl of Taqman master mix(2×, Vazym, China) with water added to give a final volume of 50μl per sample. P in the real-time PCR amplification performed on an ABI 7500 detection system in following conditions: 94°C for 2 min and 40 cycles of 94°C for 15 s and 60°C for 45s.

### SAT-MP for detection of *M*. *pneumoniae*

In the SAT technology, 16S rRNA was transcribed into cDNA equipped with the T7 promoter with the help of Moloney murine leukemia virus (MLV) reverse transcriptase and then multiple RNA copies(100 ~ 1000) were produced from each cDNA copy by use of T7 RNA polymerase. Afterwards, these RNA copies were transcribed into cDNAs again and bound to fluorescence-labeled specific probes to generate fluorescence [11]. 400μl of the mixture containing sample and RNA protective agent was moved into a 1.5-ml microcentrifuge tube and then added 100μl of nucleic acid extraction. RNA extraction was performed by magnetic beads according to the manufacturer’s instructions (Shanghai Rendu Biotechnology Co, Ltd). The RNA was eluted with 40μl detection reagent (dNTP, NTP, buffer, 500 nM each of primers, 250 nM of probes, internal control, Shanghai Rendu Biotechnology Co, Ltd). Then 30μl RNA sample and 10μl enzyme reagent (MLV and T7, Shanghai Rendu Biotechnology Co, Ltd) were mixed as 40μl final detection system. The primers and probes were shown in Table 1. In our study, SAT-MP assay was performed on an ABI 7500 detection system in following conditions: 40 cycles of 42°C for 60s.

**Table 1:** The primers and probes used in SAT-MP.

Primer 1	5’AATTTAATACGACTCACTATAGGGAGACACCGCTCCACATGAAATTCCAAAACTCCC3’
Primer 2	5’CGGTAATACATAGGTCGCAAGC3’
MP-probe	FAM-5’CGGACUAUUAAUCUAGAGUGUGUCCG3’-DABCYL
Internal control-probe	HEX-5’ CCGACAGUACAGCUGAGACCACUUUGAUAGUCGG3’-DABCYL

### Statistical analysis

The results were analyzed using SPSS software (version 20.0). Mc Nemar’s test and kappa test with the continuity correction were performed to analyze the relationship of serological test, real-time PCR and SAT. Two-tailed P value of less than 0.05 was considered to be statistically significant and kappa value greater than 0.75 was considered as perfect agreement.

## Result

### The capacity of SAT-MP assay

**Fig 1:**
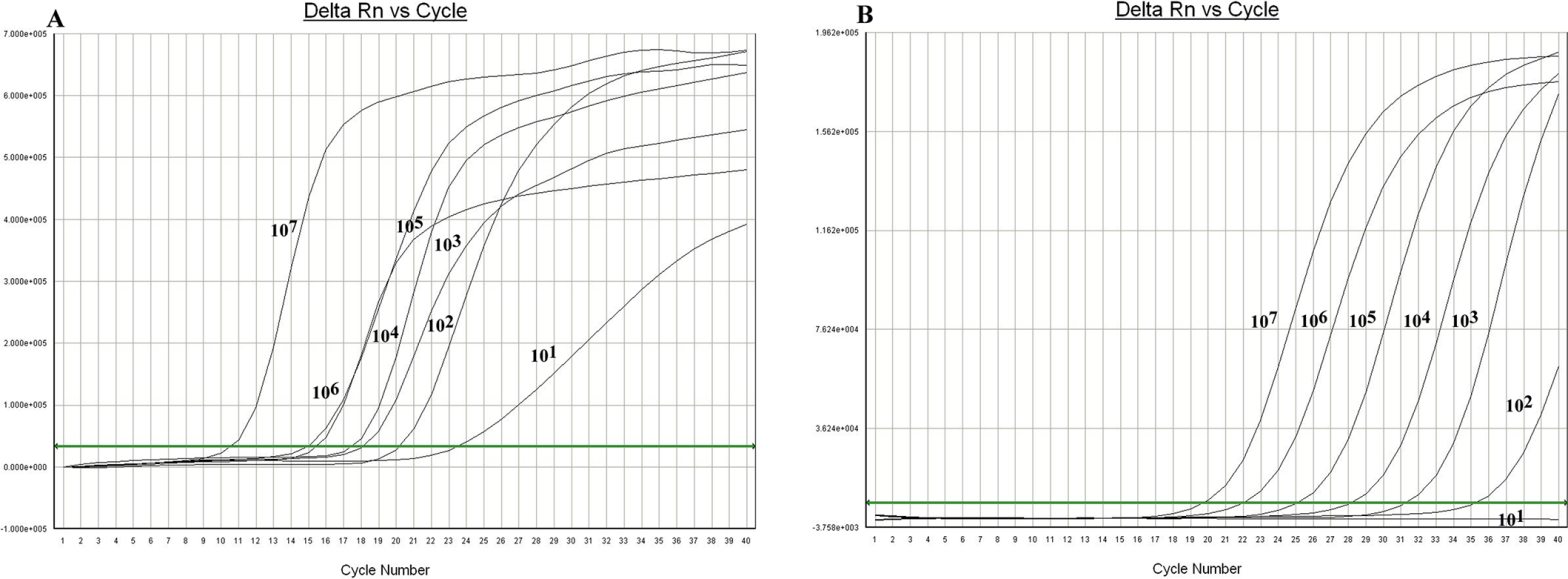
The results of sensitivity tests (10^7^CFU/ml-10^1^CFU/ml). A. SAT-MP assay B. real-time PCR.

To determine the detection range of SAT-MP assay, a serial 10-fold dilution from 10^1^ to 10^7^ CFU /ml of standard *M*. *pneumoniae* M129 (ATCC 29342) was prepared which were quantified by the commercial real-time PCR kit (Da’an gene company, China). As shown in Fig 1, all specimen were found to be *M*. *pneumoniae* positive in the SAT-MP assay. Meanwhile, the real-time PCR assay indentified specimen of which *M*.*pneumoniae* concentrations ranged from 10^2^ to10^7^CFU/ml, indicating that real-time PCR methodwas not sensitive enough to detect 10^1^ CFU/ml of *M*. *pneumoniae*.

**Fig 2:**
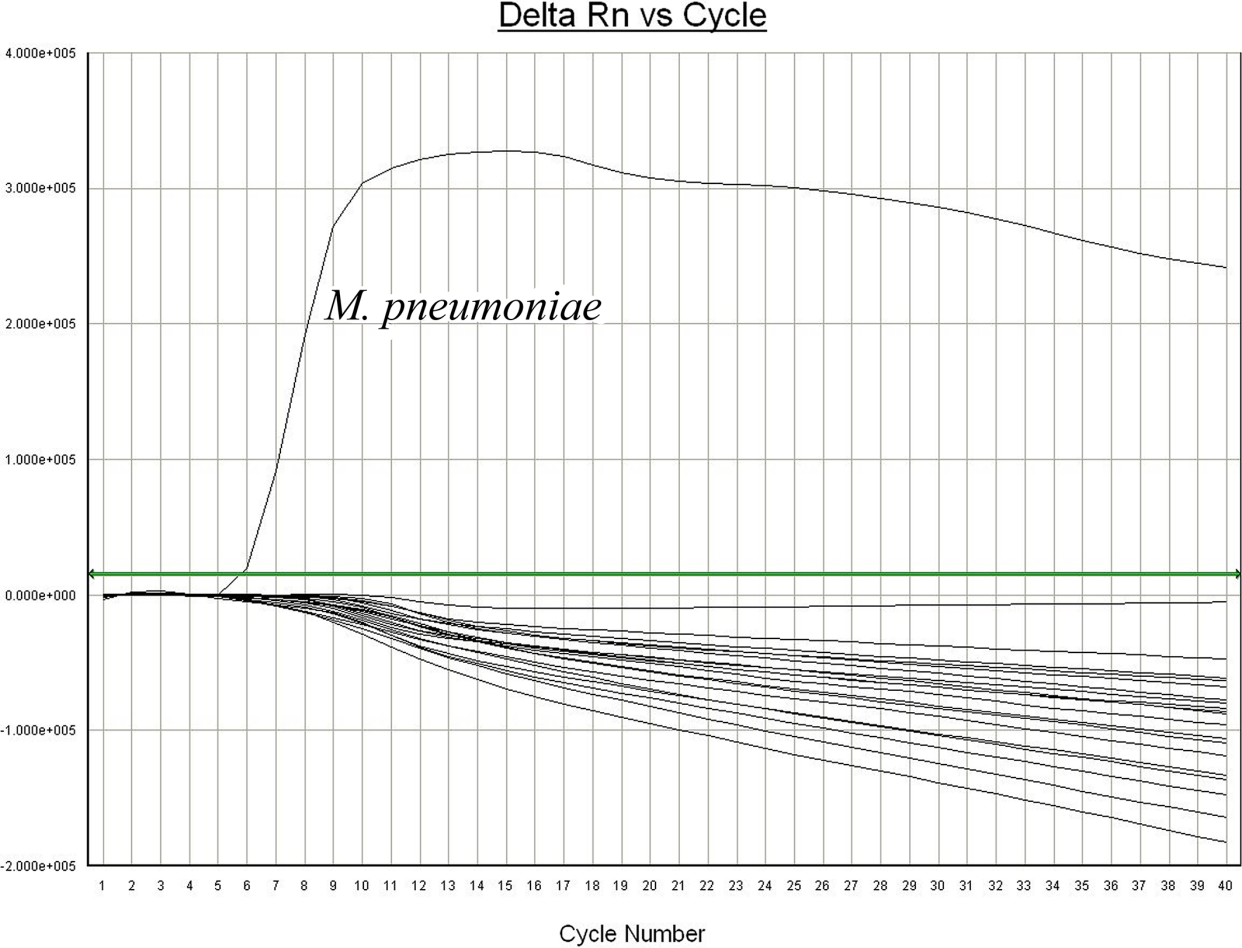
The results of specificity tests.

To assess the specificity of SAT-MP assay, a total of 18 clinical pathogens were also tested by this method, including *Escherichia coli*, *Staphylococcus aureus*, *Streptococcus pneumoniae*, adenovirus, *Klebsiella pneumoniae*, *Legionella pneumophila*, *Haemophilus influenzae*, *Pseudomonas aeruginosa*, *Chlamydia trachomatis*, *Ureaplasma urealyticum*, respiratory syncytial virus, parainfluenza virus, influenza virus, human cytomegalovirus, human metapneumovirus, enteroviruses, *Mycoplasma salivarium*, *Mycoplasma amphoriforme* and *Mycoplasma orale*. Our results showed that *M*. *pneumoniae* was identified specifically via SAT-MP assay (Fig 2).

### Data of clinical specimens

During study period, double samples were collected from every child and tested by SAT-MP assay and real-time PCR method respectively. A total of 315 samples including 197 throat swabs and 128 sputum specimens were tested by SAT-MP assay and 315 identical specimens were tested by real-time PCR method at the same time. Meanwhile, blood samples from each child were identified by *M*. *pneumoniae* IgM antibody detection assay. As shown in Table 2, among the samples from children with pneumonia, 52(16.51%) of them were positive for *M*. *pneumoniae* by using SAT-MP assay and 49(15.56%) were positive tested by real-time PCR method which were also positive in SAT-MP assay. There was no statistical significance (*p* = 0.25) between SAT-MP assay and real-time PCR method. But a high statistical consistency (kappa value = 0.97) were observed between the two assays. The Ct value of 3 negative samples in real-time PCR method were 20.46, 23.22 and 22.16 by SAT-MP assay respectively.

**Table 2:** The data of specimens from children with pneumonia.

SAT-MP assay	Real-time PCR method		Total	IgM assay		Total
	Positive	Negative		Positive	Negative	
Positive	49	3	52	38	14	52
Negative	0	263	263	2	261	263
Total	49	266	315	40	275	315

Blood samples of all children were also collected and tested by *M*. *pneumoniae* IgM assay. Among 315 samples, 40 (12.58%) were found positive via IgM assay, 38 of which were also positive in SAT-MP assay and real-time PCR method. Results of SAT-MP were significantly different (*p* value = 0.04) from those of IgM assay. As for the 3 negative samples in real-time PCR method which were mentioned above, two of them were positive and one was negative in IgM assay.

## Discussion

*M*. *pneumoniae* is a major cause of pneumonia in children and required rapid, sensitive and specific detection in clinical practice [17]. Our previous study found that immunochromatographic antigen assay could support fast diagnosis of Mycoplasma pneumoniae infection with limitied sensitivity [18]. Real-time PCR test is frequently used for identification of *M*. *pneumoniae* in clinical practice for its high sensitivity and specificity [19,20]. In the present study, we evaluated the potential utility of SAT-MP assays in the diagnosis of *M*. *pneumoniae* infection in children with pneumonia. The SAT-MP assay targeting to rRNA of *M*. *pneumoniae* is much more labile than DNA due to fast decomposition of RNA [13, 14]. Of note, SAT-MP amplification products are also RNA, suggesting a reduced risk of laboratory contamination and false-positive results [14]. We found that SAT-MP assay was more sensitive to *M*. *pneumoniae* than real-time PCR with a detection limit of 10^1^ CFU /ml. This superior sensitivity may be attributed to higher level of rRNA than DNA at the same concentrations of *M*. *pneumoniae*. Moreover, the amplification kinetics of SAT-MP is more efficient than PCR (SAT-MP can produce 10^2^−10^3^ products in one reaction). SAT-MP also has desirable specificity in identifying *M*. *pneumoniae* with no cross-reactions seen in other pathogens.

In clinical practice, SAT-MP assay exhibited desirable performance to diagnose *M*. *pneumoniae* infection in children with pneumonia. Among 315 study participants, 52 samples were found *M*. *pneumoniae* positive via SAT-MP and 49 samples were found positive via real-time PCR. A high statistical consistency (kappa value = 0.97) was found between the SAT-MP assay and real-time PCR method. In our study, 3 SAT-MP positive samples were found negative in real-time PCR and the Ct value of them in SAT-MP assay were higher than 20. Given the superior sensitivity of SAT-MP, it is reasonable to presume that the concentrations of *M*. *pneumoniae* in these three specimens may be lower than the detection limit of real-time PCR. In addition, only 40 samples were shown positive results by IgM assay and the positive rate was significantly lower than that of SAT-MP. Because IgM to *M*. *pneumoniae* did not appear at the initial stage infection. It is critical to apply new rapid diagnostic tools such as SAT to improve early diagnosis and timely treatment in clinical application. Moreover, the SAT-MP assays can be completed in only 2–3 hours, and the cost of SAT-MP assay was 20 US dollars in Zhejiang province.

In conclusion, SAT-MP is a rapid, sensitive and specific method for the identification of *M*. *pneumoniae*. SAT-MP would be an effective and valuable diagnostic tool for clinicians to detect *M*. *pneumoniae* at the initial phase of infection. Application of such novel technique may impact clinical practice and improve management of pneumonia in children.
